# A possible explanation for resistance in schizophrenia

**DOI:** 10.1192/j.eurpsy.2022.1177

**Published:** 2022-09-01

**Authors:** B. Emna, A. Larnaout, K. Souabni, R. Lansari, W. Melki

**Affiliations:** 1 military hospital of Tunis, Psychiatric Departement, Denden, Tunisia; 2 Razi Hospital, Psychiatry D, Manouba, Tunisia

**Keywords:** arachnoid cyst, resistant schizoaffective disorder, comorbidity with schizophrenia

## Abstract

**Introduction:**

Arachnoid cyst is a neurological tumor. It’s rare and benign. Its association to psychosis has been described in literature.

**Objectives:**

Through a case report and a review of the literature we hypothesize that arachnoid cyst is the cause of resistance in a patient with schizoaffective disorder.

**Methods:**

Starting from a case report, we conducted a literature review on “PubMed”, using key words “arachnoid cyst”, “arachnoid cyst a psychiatry”, “arachnoid cyst and schizoaffective disorder”, “arachnoid cyst and schizophrenia”

**Results:**

Mr. AA is 50 years old, has diabetes treated with metformin, hypercholesterolemia and celiac disease under gluten free diet. He has been diagnosed with schizoaffective disorder in 1992, initially put on haloperidol and carbamazepine. Since the patient wasn’t getting better, we suspected no-compliance so we switched haloperidol for fluphenazine decanoate. The patient still suffered from persecutory delusion and auditory hallucinations. We started him on clozapine still with no improvement. So, we concluded to the resistance of schizoaffective disorder considered electroconvulsive therapy (ECT). A cerebral MRI was conducted, prior to ECT, objectifying a left anterior frontal arachnoid cyst of 26 millimeters from the main axis producing a mass effect on the cerebral cortex. This neurological tumor didn’t require neurosurgery.

**Conclusions:**

Our patient was resistant to all treatments including clozapine. The only anomaly discovered was the arachnoid cyst. Could this explain the resistance of this patient and others like him? Could this be an interesting research path to further elucidate the mystery of metal disorder?

**Disclosure:**

No significant relationships.

